# Correction: Porous conducting polymer and reduced graphene oxide nanocomposites for room temperature gas detection

**DOI:** 10.1039/c8ra90047g

**Published:** 2018-06-04

**Authors:** Yajie Yang, Xiaojie Yang, Wenyao Yang, Shibin Li, Jianhua Xu, Yadong Jiang

**Affiliations:** State Key Laboratory of Electronic Thin Films and Integrated Devices, School of Optoelectronic Information, University of Electronic Science and Technology of China (UESTC) Chengdu 610054 P. R. China jj_eagle@163.com +86-28-83206123 +86-28-83208959

## Abstract

Correction for ‘Porous conducting polymer and reduced graphene oxide nanocomposites for room temperature gas detection’ by Yajie Yang *et al.*, *RSC Adv.*, 2014, **4**, 42546–42553.

The authors wish to draw the reader’s attention to their previous related study, published in *ACS Applied Materials & Interfaces*,^1^ which was not cited in this *RSC Advances* paper. The *RSC Advances* paper is a valuable and necessary supplement to the publication in *ACS Applied Materials & Interfaces* and focused on the reduced gas sensing performance of the devices instead of film characterization and film conductive performance.

The authors regret not giving correct attribution to Fig. 1–6 which duplicate data from ref. 1 and therefore should be attributed to that paper. Additionally, there are portions of overlapping text in the discussion of these figures and the Introduction and Conclusion sections of the *RSC Advances* paper, which should be attributed to ref. 1.

The Royal Society of Chemistry apologises for these errors and any consequent inconvenience to authors and readers.

## Supplementary Material
